# Clinical Analysis of Single and Double Sacroiliac Screws in the Treatment of Tile C1 Pelvic Fracture

**DOI:** 10.1155/2022/6426977

**Published:** 2022-01-04

**Authors:** Hong-Li Deng, Dong-Yang Li, Yu-Xuan Cong, Bin-Fei Zhang, Jin-Lai Lei, Hu Wang, Peng-Fei Wang, Yan Zhuang

**Affiliations:** ^1^Department of Orthopedic Trauma, Honghui Hospital, Xi'an Jiaotong University, Xi'an, 710000 Shaanxi, China; ^2^Xi'an Medical University, Xi'an, 710000 Shaanxi, China

## Abstract

We investigated the difference between fixation of single and double sacroiliac screws in the treatment of Tile C1 pelvic fractures. The data of 54 patients with Tile C1 pelvic fractures who were admitted to the trauma center of the Red Society Hospital Affiliated to Xi'an Jiaotong University between August 2016 and August 2020 were retrospectively analyzed. All patients with posterior pelvic ring injuries underwent fixation with sacroiliac screws assisted by a percutaneous robotic navigation system. The operative time, amount of intraoperative blood loss, and postoperative follow-up time between the two groups (single sacroiliac and double sacroiliac screw groups) were compared. The Matta and Majeed scores at the last follow-up were compared between the groups to evaluate fracture reduction and functional recovery. Forty-nine patients were followed up for 17.2 (±4.5) months and 16.2 (±3.4) months in the single and double sacroiliac screw groups, respectively. All patients had excellent fracture reduction immediately after surgery, according to the Matta score. All fractures healed without complications. There was no statistically significant difference in preoperative general information, amount intraoperative blood loss, intraoperative anterior ring fixation method, and postoperative follow-up time between the two groups (*P* > 0.05). The operative time of the single sacroiliac screw group was shorter than that of the double sacroiliac screw group (*P* < 0.05). At the last follow-up, the Matta score of the double sacroiliac screw group was significantly better than that of the single sacroiliac screw group (*P* < 0.05), and there was no statistically significant difference in the Majeed functional scores (*P* > 0.05). For Tile C1 pelvic fractures, double sacroiliac screw fixation of posterior ring injuries can provide a more stable treatment with no statistically significant difference in functional recovery.

## 1. Introduction

Pelvic fracture is a serious injury that is most often caused by high-energy trauma, such as falling from a height or car accidents, with many combined injuries [1] and a certain risk of death [2]. Tile C1 pelvic fracture mainly involves unilateral pelvic ring instability, which is a serious type of pelvic injury. Patients with unsatisfactory reduction and fixation often experience pain, deformity, and lower limb dysfunction. For the treatment of Tile C1 posterior pelvic ring injury, open reduction and internal fixation can achieve good efficacy, but the trauma is severe, and the amount of intraoperative bleeding is considerable. Percutaneous sacroiliac screws can effectively solve the aforementioned problems and are a reliable method to fix the instability of the posterior pelvic ring [3, 4].

Accurate sacroiliac screw placement has long since been a major research topic. With the development of digital orthopedics, a variety of auxiliary screw placement technologies have emerged to enable precise sacroiliac screw placement. Gandhi et al. demonstrated that CT-guided SI joint stabilization offers many advantages, including safe and accurate screw placement, reduced operating time, early definitive fixation, immediate mobilization, and fewer infections and wound complications [5]. Takeba et al. also achieved satisfactory clinical results using O-arm-assisted nail placement technology [6]. Florio et al. showed that 3D navigation significantly improved the accuracy of sacroiliac screw placement [7].

Currently, the number and length of sacroiliac screws are the primary foci for research. Yinger et al. [8] and Van Zwienen et al. [9] concluded that double sacroiliac screws should be used whenever possible for unilateral unstable sacral fractures. Cavalcanti Kußmaul et al. [10] showed that double sacroiliac screws had higher biomechanical stability than single sacroiliac screws in a model of C1.3 pelvic fracture in vitro. However, Sagi et al. [11] showed that adding multifaceted sacroiliac screws did not significantly increase the stability of the hemipelvis. Previous studies focused on biomechanical investigations of sacroiliac screw fixation. We aimed to explore whether fixation of the posterior pelvic ring with single versus double sacroiliac screws would affect the clinical outcomes of patients with Tile C1 pelvic fractures.

## 2. Materials and Methods

### 2.1. Inclusion and Exclusion Criteria

The inclusion criteria were as follows: (1) acute Tile C1 pelvic fracture; (2) amenable conditions for screw placement, with all screw placements being common screws; and (3) postoperative fracture reduction being satisfactory.

The exclusion criteria were as follows: (1) old pelvic fractures (>3 weeks); (2) bilateral sacroiliac joints being penetrated by the insertion screw; (3) severe cardiopulmonary, hepatic, and renal incompetence or coagulation dysfunction; (4) inability to undergo surgery within 3 weeks due to open injury, infection, or combined injury; and (5) poor postoperative fracture reduction.

### 2.2. General Information

A total of 54 patients were included in this study. All patients with posterior pelvic ring injuries underwent fixation with sacroiliac screws assisted by a percutaneous robotic navigation system. The patients were divided into two groups based on the number of sacroiliac screws implanted. The single sacroiliac screw group had 24 patients (17 men and 7 women), with an average age of 41.4 (±11.6) years. Side analysis: ten patients were on the left side, and 14 patients were on the right side. Body mass index (BMI) was 24.1 (±1.9) kg/m^2^. The causes of injury were traffic accidents (15 cases) and fall from a height (9 cases). Ten patients had complicated internal diseases. According to the Tile classification, two cases were of type Tile C1.1, seven were of type Tile C1.2, and 15 were of type Tile C1.3. The time from injury to operation was 6.75 (±1.7) days. In the double sacroiliac screw group, there were 30 patients (20 men and 10 women), aged 44.7 (±11.8) years on average. Side differences: fourteen patients were on the left side, 16 patients were on the right side. BMI was 23.6 (±1.8) kg/m^2^. The causes of injury were traffic accidents (19 cases) and fall from a height (11 cases). Twelve patients had complicated internal diseases. According to the Tile classification, there were three cases of Tile C1.1, six cases of Tile C1.2, and 21 cases of Tile C1.3. The time from injury to operation was 6.48 (±1.2) days. This study was approved by the Medical Ethics Committee of the Red Society Hospital Affiliated to Xi'an Jiaotong University (No. 2020124).

### 2.3. Treatment Methods

#### 2.3.1. Preoperative Preparation

After admission, patients with combined injuries were treated accordingly to maintain the stability of vital signs. The supracondylar bone of the affected side of the femur was used to perform traction in all patients, and the maximum traction weight could be up to 1/5th of patient's body weight. The traction weight should be personalized according to patient's condition, and the weight should be gradually increased to the maximum traction weight. For elderly patients with osteoporosis, the bone traction position should be slightly higher, to enable easier to adapt to a larger weight. In addition to considering the influence of the weight of the limbs and the friction of the skin and bed surface on the traction weight, the influence of the muscle tension of patients should also be considered. Among people with the same weight, the traction weight of people with more developed muscles should be greater. The operative timing was selected after patient's vital signs were stabilized. A routine preoperative enema was performed to drain intestinal gas. Pelvic anteroposterior and inlet and exit radiographs, plain CT scan of the pelvis, and three-dimensional reconstruction were also performed.

#### 2.3.2. Intraoperative Operating

**Figure 1:**
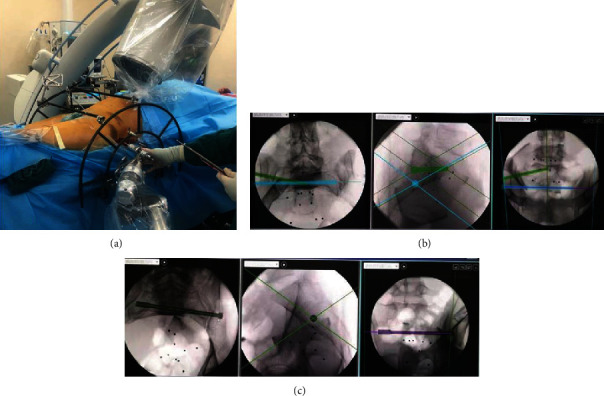
Preoperative traction fracture displacement was not corrected, and the intraoperative reduction was assisted by Starr frame and screw implantation assisted by robotic navigation system. (a) Starr frame-assisted reset. (b) Intraoperative planning of double sacroiliac screws was performed by a robotic navigation system. (c) Intraoperative planning of a single sacroiliac screw was performed with a robotic navigation system.

In all cases, posterior pelvic ring injuries were fixed with a half-threaded hollow screw with an AO diameter of 7.3 mm. The patient was placed in a supine position on the orthopedic fluoroscopy bed after general anesthesia, and if the posterior pelvic ring was not completely reduced preoperatively, a Starr pelvic reduction rack was installed for reduction (Figure 1). The channel safety of screw placement was confirmed after satisfactory reduction. A single screw was inserted when the safety of the S1 or S2 channel could not be confirmed due to stenosis, deformity, unclear fluoroscopy, or other reasons. Otherwise, two screws were inserted. Robotic navigation systems were used in all patients to assist in fixation of the posterior pelvic ring. After satisfactory reduction of the posterior ring in all patients, the robotic navigation system was used to assist in the fixation of screws in the posterior pelvic ring. The robot workstation was connected to the robot arm and a C-arm X-ray machine with data lines to start, and the sacroiliac screw to implant the surgical module and the left or right side of the surgical site were selected. The aiming sleeve was installed on the manipulator arm of the robot, and external fixation pins were placed percutaneously around the anterior superior iliac spine to install the patient tracer. Personalized fluoroscopy was performed using the C-arm to obtain the standard pelvic inlet, pelvic outlet, and lateral phases. A locator was installed, and 10 positioning targets were placed simultaneously in the field of vision obtained by C-arm fluoroscopy. The inlet, pelvic outlet, and lateral phases were collected, and nail placement was planned (Figure 1). On the premise that the screw position is safe, the robot manipulator will deliver the aiming sleeve to the designated area according to the planning instruction. Subsequently, a skin incision was made, soft tissue along the bone surface was separated, and a secondary cannula was installed. The guide wire was drilled along the catheter, the hole was reamed, and a cannulated screw was placed. C-arm fluoroscopy was performed to determine the position and length of the screw, and the guidewire was then removed.

Method of fixation of the anterior pelvic ring: in the single sacroiliac screw group, there were 15 cases of plate fixation, three cases of cannulated screw fixation, and two cases of external fixation. In the double sacroiliac screw group, 21 cases were fixed with a plate, six with a cannulated screw, and two with external fixation.

#### 2.3.3. Postoperative Management

All patients were treated with antibiotics to prevent infection and low-molecular-weight heparin to prevent deep vein thrombosis of the lower extremities. The wound was disinfected and dressed regularly, and stitches were removed two weeks after surgery. On the second day after surgery, patients were instructed to turn over, actively flex and extend the hips and knees, and contract the muscles of both lower limbs on the bed. They could sit up 2-3 weeks after surgery, walk with two crutches without weight-bearing 4-6 weeks after surgery, walk with partial weight-bearing 6-8 weeks after surgery, and walk with full weight-bearing 10-12 weeks after surgery. Regular outpatient visits were scheduled at 1, 3, 6, and 12 months for review, and then visits were scheduled every six months until 2 to 4 years after the surgery.

### 2.4. Observation Indicators

Sex, age, body mass index, injured side, cause of injury, fracture type, time from injury to operation, combined medical diseases, and other general information of patients were recorded. The operative time, amount of intraoperative blood loss, anterior ring fixation method, and postoperative follow-up time were recorded as well. Matta and Majeed scores were recorded at the last follow-up. The Matta score was used to evaluate the reduction in pelvic fractures after internal fixation. The evaluation criteria were as follows: excellent, the displacement range of the posterior pelvic ring was less than 4 mm; good, the displacement range of the posterior ring of the pelvis was 4-20 mm; and poor, pelvic posterior ring displacement was greater than 20 mm. The Majeed score mainly evaluated patients' physiological function recovery, fracture healing speed, and degree of pain. The total score of patients who worked before the injury was 100, and those who did not work was 80. After calculation, the total score was evaluated for clinical classification, and the scoring standard for patients who worked before injury was as follows: excellent score, >85; good, 70-84 points; middle, 55-69 points; or poor, less than 55 points. In patients who did not work before injury, the scores were: excellent score: >70; good, 55-69 points; middle, 45-54 points; or poor, less than 55 points.

### 2.5. Statistical Analysis

Statistical analysis was performed using IBM SPSS 23.0, and the Shapiro-Wilk test was used to determine whether the data were normally distributed. Age, time from injury to operation, amount of blood loss, and follow-up time were normally distributed with homogenous variance. An independent-sample *t*-test was used to compare numerical variables, and the chi-square test was used to compare the categorical data. Statistical significance was set at *P* < 0.05.

## 3. Results

**Figure 2:**
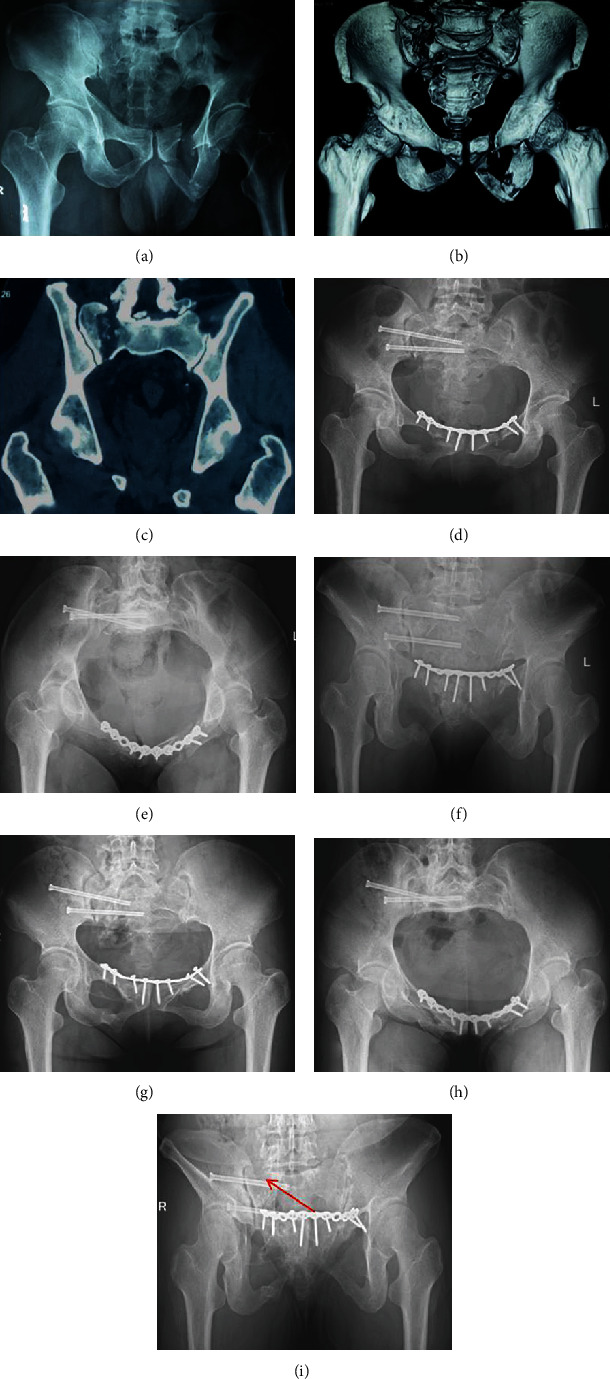
Patient, female, 46, pelvic fractures, front ring plate fixation. The posterior ring was fixed with double sacroiliac screws. (a) Anteroposterior view of pelvic fracture. (b) 3D CT preoperative pelvic fractures for Tile C1.3 type, unilateral vertically unstable pelvic ring. (c) Preoperative CT scan in pelvic posterior ring by the sacrum fracture, vertically displaced. (d) Anteroposterior radiograph of the pelvis immediately after the operation showed satisfactory reduction of the fracture. (e) Inlet radiograph of the pelvis immediately after surgery. (f) Outlet radiograph of the pelvis immediately after surgery. (g) Anteroposterior radiographs of the pelvis 20 months after surgery showed fracture healing. (h) A radiograph of the pelvic inlet 20 months after surgery showed fracture healing. (i) A radiograph at the exit of the pelvis 20 months postoperatively showed healing of the fracture and no loss of reduction at the arrow.

**Figure 3:**
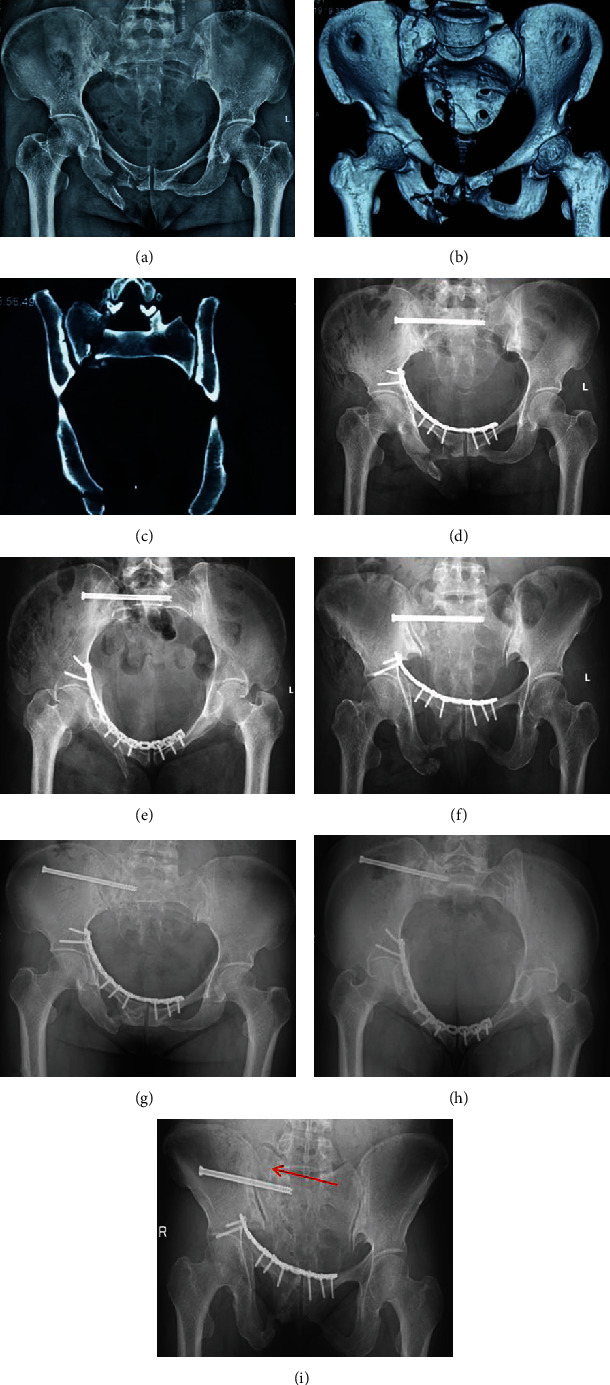
Patient, female, 41, with pelvic fractures, front ring plate fixation. The posterior ring was fixed with a single sacroiliac screw. (a) Anteroposterior view of pelvic fracture. (b) 3D CT preoperative pelvic factures for Tile C1.3 type, unilateral vertically unstable pelvic ring. (c) Preoperative CT scan in pelvic ring through the sacrum fracture, vertically displaced. (d) Anteroposterior radiograph of the pelvis immediately after the operation showed satisfactory reduction of the fracture. (e) Inlet radiograph of the pelvis immediately after surgery. (f) Outlet radiograph of the pelvis immediately after surgery. (g) Anteroposterior radiographs of the pelvis 18 months after surgery showed fracture healing. (h) A radiograph of the pelvic inlet 18 months after surgery showed fracture healing. (i) A radiograph at the exit of the pelvis 18 months postoperatively showed healing of the fracture and loss of reduction at the arrow.

Among all of the patients who initially participated, one did not complete follow-up due to incomplete clinical data, two were lost to follow-up, and two underwent revision surgery after failure of internal fixation; thus, 49 patients were followed up for 17.2 (±4.5) months in the single sacroiliac screw group and 16.2 (±3.4) months in the double sacroiliac screw group. All patients had excellent fracture reduction immediately after surgery, according to the Matta score. The fat in the wound of one patient was liquefied after surgery, and the wound healed after repeated dressing changes, and another patient had screw withdrawal. All fractures healed clinically without infection, fracture nonunion, delayed union, internal fixation fracture, or other complications. Typical cases are shown in Figures 2 and 3, respectively.

There were no significant differences in preoperative general information, such as sex, age, side type, injury type, combined medical diseases, and time from injury to operation between the two groups (*P* > 0.05) (Table 1). There were no statistically significant differences in amount of intraoperative blood loss, intraoperative anterior ring fixation method, and postoperative follow-up time between the two groups (*P* > 0.05) (Table 2). The operative time in the single sacroiliac screw group was shorter than that in the double sacroiliac screw group (*P* < 0.05) (Table 2). At the last follow-up, the Matta score of the double sacroiliac screw group was significantly better than that of the single sacroiliac screw group (*P* < 0.05) (Table 3). There was no statistically significant difference in the Majeed functional scores (*P* > 0.05) (Table 4).

## 4. Discussion

### 4.1. Status of Treatment of Tile C1 Pelvic Fracture

The anterior and posterior rings of Tile C1 pelvic fractures are injured, and both the rotation and vertical directions are unstable [12]. Since the anterior pelvic ring structure accounts for 40% of the stabilization of the pelvis and the posterior pelvic ring structure accounts for 60%, the focus of internal fixation should be placed on the posterior pelvic ring [13]. Commonly used fixation methods include plate fixation, sacral rod fixation, and sacroiliac screw fixation. Percutaneous sacroiliac screw fixation of the posterior pelvic ring has the advantages of stabilizing the pelvis at an early stage, reducing local soft tissue scarring, minimizing the injury, and leading to less blood loss [14]. Therefore, sacroiliac screws have become a commonly used, minimally invasive technique for the fixation of unstable posterior pelvic ring injuries [15]. However, clinical studies have shown that not all sacroiliac screw fixations can achieve stability. Keating et al. [16] used sacroiliac screws to fix vertically unstable posterior pelvic ring injuries. Although an 84% anatomical reduction or near-anatomical reduction rate was achieved, the final follow-up showed that the malunion rate was as high as 44%. Griffin et al. [17] studied 62 cases of vertically unstable pelvic posterior ring injuries with sacroiliac screw fixation. They found that single sacroiliac screws led to fixation failure and were more likely to lose resets. Therefore, the question is how to maximize the biomechanical stability of the sacroiliac screw, reduce the failure rate of fixation, and improve its clinical curative effect. This is particularly important in the treatment of Tile C1 pelvic fractures.

### 4.2. Application of Sacroiliac Screw in Tile C1 Pelvic Fracture

Single sacroiliac screw fixation refers to the fixation of the posterior pelvic ring at a single level of S1 or S2, while double sacroiliac screw fixation refers to the fixation of the posterior pelvic ring at both levels of S1 and S2. Currently, a large body of biomechanical research shows that using two screws to prevent the ilium from revolving around single pieces of sacroiliac screws can improve the stability of the fixed pelvic ring. Moreover, orthopedics robot navigation technology is being increasingly used in the treatment of pelvic fractures. These systems can accurately place screws, avoid the possibility of neurovascular injury, and reduce the risk associated with the placement of two sacroiliac screw [18, 19]. Therefore, some surgeons tend to choose two sacroiliac screws to fix pelvic posterior rings. The choice of sacroiliac screw placement for osteoporosis patients or even through the bilateral sacroiliac joints. However, penetration of the bilateral sacroiliac screws can destroy the anatomical structure of the sacroiliac joint plane on the healthy side, causing irreversible effects on the physiological function of the sacroiliac joint and increase the possibility of nerve injury. Therefore, for unilateral pelvic posterior ring injuries without osteoporosis, penetration of the bilateral sacroiliac joints is generally not used for fixation. The stability of the anterior ring also affects the selection of posterior ring fixation methods for the treatment of unstable pelvic fractures. Cavalcanti Kußmaul et al. [10] investigated different fixing ways to treat type Tile C1.3 pelvic fractures in biomechanical studies and showed that the two sacroiliac screws combined with retrograde pubic ramus screws achieved minimal displacement and the greatest stability, while external fixation combined with a single sacroiliac screw had the worst stability. Therefore, when the stability of anterior ring fixation is poor, multiplanar sacroiliac screws should be selected for posterior ring. When the stability of anterior ring fixation is reliable, a single screw can be selected for fixation.

### 4.3. Experience and Analysis of Sacroiliac Screw in Tile C1 Fracture of Pelvis

In this study, the efficacy of single and double sacroiliac screws in the treatment of Tile C1 type pelvic fractures was compared. The results showed that the operation time of single sacroiliac screw fixation was shorter than that of double sacroiliac screw fixation (*P* < 0.05) and that the Matta score at the last follow-up between the two screws was statistically significant (*P* < 0.05), while the Majeed score was not statistically significant (*P* > 0.05). The short operative time of the single sacroiliac screw group was mainly attributed to the number of screws inserted and the proficiency of using the robotic navigation system. Based on the results of the analysis, a single sacroiliac screw did not provide a satisfactory reduction effect for Tile C1 type fractures, which is similar to the results of previous biomechanical studies. However, compared with double sacroiliac screw fixation, a single sacroiliac screw, despite loss of reduction during fracture healing, did not affect patients' functional recovery and long-term quality of life. This is different from the results reported in other studies. Muzii et al., in their study on the impact of reduction results on spinal and pelvic balance, gait, and quality of life in complex pelvic acetabular fractures, showed that incomplete reduction may lead to secondary spino-pelvic imbalance accompanied by posture and gait disorders, resulting in poor functional rehabilitation results [20]. Therefore, a single screw was inserted for the treatment of Tile C1 type pelvic fractures when the safety of the S1 or S2 channel could not be confirmed due to stenosis, deformity, unclear fluoroscopy, or other reasons. In addition, the use of two sacroiliac screws is undoubtedly a better choice, especially for patients with osteoporosis or where the anterior ring cannot be effectively fixed. The indications for the two sacroiliac screws should be carefully evaluated in future studies or clinical practice to determine which patients could truly benefit from it.

### 4.4. Limitations of This Study

The sample size was small and it was a single-center study. The follow-up time was short, and long-term efficacy could not be further evaluated. A high rate of missed follow-up leads to an overestimation of the therapeutic efficacy [21]. Pelvic fractures are often associated with other fractures, which may affect the overall functional score and the results.

## 5. Conclusions

For Tile C1 pelvic fractures, double sacroiliac screw fixation of posterior ring injuries can provide a more stable treatment, but there is no statistically significant difference in functional recovery when compared that obtained with single sacroiliac screw fixation.

## Figures and Tables

**Table 1:** Comparison of preoperative general data.

Indicators	Single screw	Double screws	Statistics	*P* values
Sex (male/female)	17/7	21/9	0.113	0.737
Age (years, **x** ± **s**)	41.4 ± 11.6	44.7 ± 11.8	0.172	0.340
Injury cause (traffic/fall)	15/9	19/11	0.165	0.684
Classification (C1.1/C1.2/C1.3)	2/7/15	3/6/21	1.531	0.148
Side (left/right)	10/14	14/16	1.637	0.201
Body mass index (BMI) (kg/m^2^, **x** ± **s**)	24.1 ± 1.9	23.6 ± 1.8	0.154	0.378
Time from injury to operation (h, **x** ± **s**)	6.75 ± 1.7d	6.48 ± 1.2d	0.173	0.462
Complicated internal diseases (yes/no)	10/14	12/18	0.694	0.155

**Table 2:** Comparison of operative time, amount of blood loss, and follow-up time.

Indicators	Single screw	Double screws	Statistics	*P* values
Operation time (h, *x* ± *s*)	3.4 ± 1.3	4.4 ± 0.8	6.014	0.01
Blood loss (ml, *x* ± *s*)	485.0 ± 161.5	513.8 ± 203.93	0.809	0.61
Follow-up time (month, *x* ± *s*)	17.2 ± 4.5	16.2 ± 3.4	1.400	0.350
Front ring fixation (plate/screw/external fixation)	15/3/2	21/6/2	1.300	0.256

**Table 3:** Comparison of Matta scores at the last follow-up.

Group	Number of cases	Matta score			
		Optimal	Good	Middle	Poor
Single screw	20	7	7	4	2
Double screws	29	25	2	1	1
*T* value	—	9.67			
*P* values	—	0.012			

**Table 4:** Comparison of Majeed scores at the last follow-up.

Group	Number of cases	Majeed score			
		Optimal	Good	Middle	Poor
Single screw	20	14	5	1	0
Double screws	29	15	13	1	0
*T* value	—	2.22			
*P* values	—	0.351			

## Data Availability

The data used to support the findings of this study are available from the corresponding author upon request.

## Abbreviations

BMI: Body mass index.
